# Short Message Service Text Message Support for Weight Loss in Patients With Prediabetes: Pragmatic Trial

**DOI:** 10.2196/12985

**Published:** 2019-04-15

**Authors:** Henry H Fischer, Michael J Durfee, Silvia G Raghunath, Natalie D Ritchie

**Affiliations:** 1 Denver Health and Hospital Authority Denver, CO United States; 2 Department of Psychiatry, University of Colorado School of Medicine Aurora, CO United States; 3 College of Nursing, University of Colorado Aurora, CO United States

**Keywords:** eHealth, prediabetes, texting, weight loss

## Abstract

**Background:**

To reach all 84.1 million US adults estimated to have prediabetes warrants need for low-cost and less burdensome alternatives to the National Diabetes Prevention Program (NDPP). In a previous randomized controlled trial, we demonstrated the efficacy of a 12-month short message service text message support program called SMS4PreDM amongst individuals with prediabetes.

**Objective:**

The study aimed to evaluate the implementation and effectiveness of SMS4PreDM in a pragmatic study following dissemination in a safety net health care system.

**Methods:**

English- and Spanish-speaking patients at risk for diabetes (eg, glycated hemoglobin 5.7-6.4) were referred by their providers and offered either NDPP classes, SMS4PreDM, or both. This analysis focuses on weight change among 285 SMS4PreDM-only participants who began the year-long intervention between October 2015 and April 2017 with accompanying pre- and postweights, as compared with 1233 usual-care control patients at risk for diabetes, who were identified from electronic health records during this time but not referred. Weight outcomes included time-related mean weight change and frequency of either ≥3% weight loss or gain. Mixed linear models adjusted for age, gender, race, ethnicity, preferred language, and baseline weight. A secondary analysis was stratified by language. We also assessed implementation factors, including retention and cost.

**Results:**

SMS4PreDM participants had high retention (259 of 285 patients or 91.0% completion at 12-months, ) and a time-related mean weight loss of 1.3 pounds (SE 0.74), compared with the control group’s slight mean weight gain of 0.25 pounds (SE 0.59; *P*=.004). Spanish-speaking SMS4PreDM participants (n=130) had a time-related mean weight loss of 1.11 pounds (SE 1.22) compared with weight gain of 0.96 pounds (SE 1.14) in Spanish-speaking controls (n=382, *P*<.001). English-speaking intervention participants (n=155) had a comparable time-related mean weight change (–0.89 pounds; SE 0.93) as English-speaking controls (n=828; 0.31 pounds gained; SE 0.62, *P*=.14). Overall, frequency of achieving ≥3% weight loss was comparable between groups (54 of 285 or 19.0% of SMS4PreDM participants [95% CI 14.8-23.9] vs 266 of 1233 or 21.6% of controls [95% CI 19.3-24.0]; *P*=.33). Nonetheless, more controls had ≥3% weight gain compared with intervention participants (337 of 1233 or 27.3% of controls [95% CI 24.9-29.9] vs 57 of 285 or 20.0% of SMS4PreDM participants [95% CI 16.8-25.1]; *P*=.01). SMS4PreDM delivery costs were US $100.92 per participant.

**Conclusions:**

Although SMS4PreDM was relatively low cost to deliver and demonstrated high retention, weight loss outcomes may not be sufficient to serve as a population health strategy.

## Introduction

### Background

To reach all 84.1 million US adults estimated to have prediabetes, low-cost and less burdensome alternatives to the NDPP are needed [1]. A 2015 review found median costs of US $417 per person for programs comparable with the NDPP [2]. A recent NDPP evaluation concluded that retention has been suboptimal [3], which may be because of the demands of year-long in-person meetings. In a previous randomized controlled trial (RCT), we demonstrated efficacy of a 12-month short message service (SMS) text message support program called SMS4PreDM in individuals with prediabetes [4]. Specifically, ≥3% weight loss was achieved by 30 of 79 or 38% of participants receiving the SMS4PreDM intervention (95% CI 27.7-49.3), compared with 17 of 81 or 21% of control group participants (95% CI 12.5-30.6; absolute difference of 17%; *P*=.02) [4]. Research suggests that such modest weight loss can prevent diabetes as every kilogram lost decreases risk by 16% [5]. Real-world NDPP implementation has appeared to result in less average weight loss (4.2% N=14,747 [3]) compared with that observed in its original RCT (4.9% N=1,079 [6], along with concerning disparities in outcomes for underserved participants [3]. Thus, it is important to similarly evaluate SMS4PreDM upon broader dissemination.

### Objectives

We evaluated implementation and effectiveness and implementation of SMS4PreDM in a pragmatic study following dissemination in a safety net health care system.

## Methods

English- and Spanish-speaking patients at risk for diabetes (eg, glycated hemoglobin 5.7-6.4) were referred by their providers and offered NDPP classes and/or SMS4PreDM. Outcomes from NDPP participants were recently published [7]. We focus here on evaluating effectiveness of SMS4PreDM. SMS4PreDM methodology has been described in detail [4]. In brief, SMS text message content was based on the NDPP curriculum and iteratively refined upon patient feedback. Messages promoted lifestyle change and modest weight loss and were delivered 6 days per week over 1 year. This analysis included 285 SMS4PreDM-only participants who began the intervention between October 2015 and April 2017, with accompanying baseline and follow-up weights available, as compared with a usual-care control group of 1233 patients at risk for diabetes and available weights, who were identified from electronic health record (EHR) data during the same time frame but not referred. Patients who became pregnant during the intervention or underwent bariatric surgery were excluded.

A repeating measure analysis used all weights available in the EHR from routine health care visits within a year of the individual’s start date for SMS4PreDM participants or matched identification date for controls, as well as 12-month weights collected by study personnel upon invitation for participants without recent clinical visits. Weight outcomes included mean time-related weight change and realization of either ≥3% weight loss or gain. Mixed linear models adjusted for age, gender, race, ethnicity, preferred language, and baseline weight. A secondary analysis stratified by language. We also assessed implementation factors, including retention and cost. The Colorado Multiple Institutional Review Board approved this program as an evaluation project, which was not registered as a clinical trial.

## Results

Participant characteristics are shown in Table 1. Retention in SMS4PreDM was high as only 20 of 285 or 7% dropped out in the first 4 weeks, with 259 of 285 or 91% completing the 12-month program. SMS4PreDM participants (n=285) had an average of 5.6 visits with weight assessments during the study period compared with 5.4 visits for the control group (n=1233; *P*=.72). Most SMS4PreDM participants (262 of 285 or 91.9%) exclusively had EHR-derived weights, with few patients having weights collected by invitation (23 of 285 or 8.1%).

The intervention group had a time-related mean weight loss of 1.3 pounds (SE 0.74), whereas the control group realized mean weight gain of 0.25 pounds (SE 0.59, *P*=.004). Spanish-speaking SMS4PreDM participants (n=130) realized time-related mean weight loss of 1.11 pounds (SE 1.22) compared with weight gain of 0.96 pounds (SE 1.14) in Spanish-speaking controls (n=382, *P*<.001). English-speaking intervention participants (n=155) did not achieve more time-related mean weight change (–0.89 pounds; SE 0.93) than English-speaking controls (n=828; 0.31 pounds gained; SE 0.62; *P*=.143). There was no significant difference between groups in frequency of reaching the ≥3% weight loss goal (54 of 285 or 19.0% of SMS4PreDM participants [95% CI 14.8-23.9] vs 266 of 1233 or 21.6% of controls [95% CI 19.3-24.0]; *P*=.33). However, more controls gained ≥3% weight compared with intervention participants (337 of 1233 or 27.3% of controls [95% CI 24.9-29.9] vs 57 of 285 or 20.0% of SMS4PreDM participants [95% CI 16.8-25.1]; *P*=.011). SMS4PreDM delivery costs were US $100.92 per participant.

**Table 1:** Demographics of short message service text message support (SMS4PreDM) and control group participants.

Demographic		Intervention (n=285)	Control (n=1233)	*P* value
**Sex, n (%)**				.003
	Female	217 (76.1)	827 (67.1)	
	Male	68 (23.9)	406 (32.9)	
**Race, n (%)**				.18
	White	218 (76.5)	895 (72.6)	
	Black	36 (12.6)	220 (17.8)	
	Asian	7 (2.5)	27 (2.2)	
	Other/unknown	24 (8.4)	91 (7.4)	
**Ethnicity, n (%)**				<.001
	Hispanic/Latino	173 (60.7)	584 (47.4)	
	Non-Hispanic/Latino	61 (21.4)	364 (29.5)	
	Unknown	51 (17.9)	285 (23.1)	
**Language, n (%)**				<.001
	English	155 (54.4)	828 (67.2)	
	Spanish	130 (45.6)	382 (30.9)	
	Unknown	0 (0)	23 (1.9)	
Age (years), mean (SD)		45.5 (12.2)	48.4 (14.6)	<.001

## Discussion

### Principal Findings

In this pragmatic study of SMS text message support for weight loss to prevent diabetes, SMS4PreDM afforded high retention at a lower cost than the NDPP. However, modest weight outcomes suggest that SMS4PreDM may not be clinically effective enough to serve as a population health strategy. Although nearly twice as many SMS4PreDM participants achieved ≥3% weight loss than controls in our previously published RCT [4], less robust weight loss outcomes were demonstrated in real-world dissemination, a trend also observed with the NDPP [3]. At the same time, fewer participants in this pragmatic trial gained ≥3% weight compared with controls (NNT 14), which may hold importance, given findings that weight gain of 1 kg/m^2^ has been shown to increase glucose dysregulation [8], although more studies of this relationship are warranted. Weight loss results of SMS4PreDM were also generally within the range of outcomes found in prior systematic reviews of mobile phone apps and SMS text messaging interventions for weight reduction [9,10].

### Limitations

Limitations include nonrandomization in our pragmatic study, which may have contributed to demographic differences observed between study groups (although analyses controlled for such factors). Analyses also excluded participants without available weights; however, conditions for weight collection were largely similar between groups overall (eg, the majority of all patients in the analysis had weights collected from approximately bimonthly, routine clinical visits). However, a potential bias is that SMS4PreDM participants who successfully lost weight may have been more likely to comply with requests for final weight measurement with study personnel. It is also unknown why Spanish-speaking individuals appear to respond better to SMS text message support than English-speaking individuals, as consistent with our prior RCT [4].

### Conclusions

Future qualitative study may be needed to help explain why SMS4PreDM and similar interventions can yield less impact upon dissemination. An educational and motivational enhancement before NDPP enrollment was shown to increase efficacy in 1 study, although potentially by excluding less activated individuals [11]. Ensuring increased readiness for weight loss may potentially improve efficacy in a population that choose SMS text message support instead of in-person classes and possibly yield greater cost-effectiveness by more appropriately matching services with patients likely to benefit from them. Further research is also needed to better understand the extent to which supplemental coaching may be necessary to optimize weight loss and which extra-visit technology-based platforms (eg, apps) are most cost-effective. Future efforts to target reach among Spanish speakers may also be merited, particularly given there is likely less-than-adequate availability of Spanish-language NDPPs or comparable in-person interventions. More broadly, SMS text messaging may be especially beneficial during the maintenance phase following weight loss, given its acceptability for long-term contact [12], coupled with modestly beneficial outcomes and a relatively low cost. Retention during the maintenance phase of the NDPP has been especially problematic [3], necessitating identification of retention-bolstering strategies. In conclusion, SMS text messaging for weight-related support may continue to hold appeal, potentially for retention purposes, and further work may be merited to determine how to incorporate its desirable features along with more clinically effective components.

## Abbreviations

EHR: electronic health record
NDPP: National Diabetes Prevention Program
RCT: randomized controlled trial
SMS: short message service
